# Full Establishment of Arbuscular Mycorrhizal Symbiosis in Rice Occurs Independently of Enzymatic Jasmonate Biosynthesis

**DOI:** 10.1371/journal.pone.0123422

**Published:** 2015-04-10

**Authors:** Caroline Gutjahr, Heike Siegler, Ken Haga, Moritoshi Iino, Uta Paszkowski

**Affiliations:** 1 Department of Plant Molecular Biology, University of Lausanne, Biophore Building, Lausanne, Switzerland; 2 Institute of Genetics, University of Munich (LMU), Biocenter Martinsried, Martinsried, Germany; 3 Botanical Gardens, Graduate School of Science, Osaka City University, Kisaichi, Katano-shi, Osaka, Japan; University of Nebraska-Lincoln, UNITED STATES

## Abstract

Development of the mutualistic arbuscular mycorrhiza (AM) symbiosis between most land plants and fungi of the *Glomeromycota* is regulated by phytohormones. The role of jasmonate (JA) in AM colonization has been investigated in the dicotyledons *Medicago truncatula*, tomato and *Nicotiana attenuata* and contradicting results have been obtained with respect to a neutral, promotive or inhibitory effect of JA on AM colonization. Furthermore, it is currently unknown whether JA plays a role in AM colonization of monocotyledonous roots. Therefore we examined whether JA biosynthesis is required for AM colonization of the monocot rice. To this end we employed the rice mutant *constitutive photomorphogenesis 2* (*cpm2*), which is deficient in JA biosynthesis. Through a time course experiment the amount and morphology of fungal colonization did not differ between wild-type and *cpm2* roots. Furthermore, no significant difference in the expression of AM marker genes was detected between wild type and *cpm2*. However, treatment of wild-type roots with 50 μM JA lead to a decrease of AM colonization and this was correlated with induction of the defense gene *PR4*. These results indicate that JA is not required for AM colonization of rice but high levels of JA in the roots suppress AM development likely through the induction of defense.

## Introduction

The phythormone jasmonic acid (JA) has long been known to be involved in defense against pests such as leaf chewing insects and pathogens such as necrotrophic microbes [1–3]. On the other hand JA and its mimick coronatine promote colonization of biotrophic pathogens by counteracting salicylic acid (SA) signaling which plays an important role in defense against biotrophic microbes and nematodes [4–6]. Therefore it has been assumed that JA might also act as a positive regulator in the development of the biotrophic relationship between plants and beneficial arbuscular mycorrhizal fungi.

The arbuscular mycorrhiza (AM) symbiosis refers to the most-widespread mutualistic association between members of 80% of examined land plant families and obligate biotrophic fungi belonging of the *Glomeromycota* [7]. The intimate interaction results from a fine-tuned signal exchange between both symbiotic partners and phytohormones, among other plant factors, are currently emerging as important regulators of AM development and the quantity of AM colonization [8–12]. AM symbioses are based on nutritional benefits for both partners: the fungi fully rely on plant-delivered carbon and plants receive mineral nutrients, most prominently phosphate, from the fungi [13]. The considerable impact of arbuscular mycorrhizal fungi (AMF) on plant mineral nutrition makes application of this symbiosis an important contribution to sustainable agriculture with reduced chemical fertilizer input [14].

Although most of our staple food derives from monocotyledons such as rice, maize or wheat the importance of JA-signaling in AM colonization of roots of these crops has so far not been addressed. However, JA levels have been found to increase in barley roots upon AM colonization [15]. Support for a role of JA signaling as a positive regulator of AM colonization was provided by a study presenting decreased AM colonization of the JA-deficient tomato mutant *suppressor of prosystemin mediated responses 2* (*spr2*) which was reversible by application of methyl-jasmonate (Me-JA) to the leaves of *spr2* [16]. In addition, antisense-suppression of the JA biosynthesis gene *ALLENE OXIDE CYLASE 1* (*AOC1*) in *Medicago truncatula* hairy roots on chimeric plants abolished the AM-mediated increase of JA-levels in roots and led to a reduction in AM colonization [17], suggesting that AM development was promoted by JA in the root hypothetically due to an elevated JA-mediated carbon-sink strength [17]. Moreover, an increase of AM colonization of *Medicago truncatula* roots was shown after repeated leaf-wounding (which induces JA-biosynthesis) or application of low concentrations of JA to the shoot [18]. The phosphate transporter gene *MtPT4*, is a quantitative marker for functional arbuscules and encodes a protein that localizes to the peri-arbuscular membrane and is responsible for the uptake of fungus-delivered phosphate [19,20]. Interestingly, increased colonization due to wounding did not only cause an increased expression of *PT4*, but also elevated shoot phosphate content as compared to mycorrhizal plants that were not subjected to wounding, indicating that wounding not only promoted AM development but also AM functionality [18]. Conversely, another study established JA-signaling as having negative effects on the extent of AM colonization because the JA-receptor mutant of tomato *jasmonate insensitive 1* (*jai*) showed higher colonization levels than wild-type. Furthermore, spraying of tomato leaves with 5 μM and 50 μM Me-JA reduced the level of root colonization significantly [21]. These data underline that the role of JA-signaling in AM interactions might depend on the plant species or growth condition [6,22].

Rice is the world’s leading staple food and an excellent and by now well-developed monocotyledon model for research on the molecular biology and genetics of AM symbiosis. Importantly, when grown under aerobic conditions rice obtains most of its phosphate via the mycorrhizal uptake pathway [23]. In the field rice might be attacked by several pests such as herbivores, nematodes or microbial pathogens for example the fungus *Magnaporthe oryzae*, (the causal agent of devastating rice blast), all of which induce JA-signaling as part of the plant defense response [24–26]. For application of AM-symbioses in rice fields it would hence be important to know the impact of JA-signaling on roots and specifically during AM symbioses. Therefore, we explored the role of JA in AM colonization of the model monocotyledon rice using the jasmonate biosynthesis deficient mutant *cpm2* [26] and exogenous application of jasmonic acid (JA).

## Results and Discussion

To functionally address the role of JA for AM colonization in rice we took advantage of the JA-deficient rice mutant *coleoptile photomorphogenesis 2* (*cpm2*), which suffers from an 11 bp deletion in the single copy gene encoding the JA-biosynthesis enzyme ALLENE OXIDE CYCLASE [26]. This deletion leads to a frameshift and therefore a change in amino acid sequence making it highly unlikely that the resulting protein can still contribute to JA biosynthesis. The *cpm2* mutant offers a unique opportunity to test the role of JA in AM development because it does not produce JA and shows classical JA-deficiency related phenotypes such as impaired fertility, perturbed photomorphogenesis and decreased resistance to *Magnaporte oryzae* [26]. By contrast, another JA-deficient rice mutant called *hebiba*, suffers from a genomic deletion of 169 kb [26,27]. This causes the loss of 26 genes including AOC, implicating the likely possibility that the deletion of other genes has an impact on AM development. Therefore the *hebiba* mutant was not used in this study.

**Fig 1:**
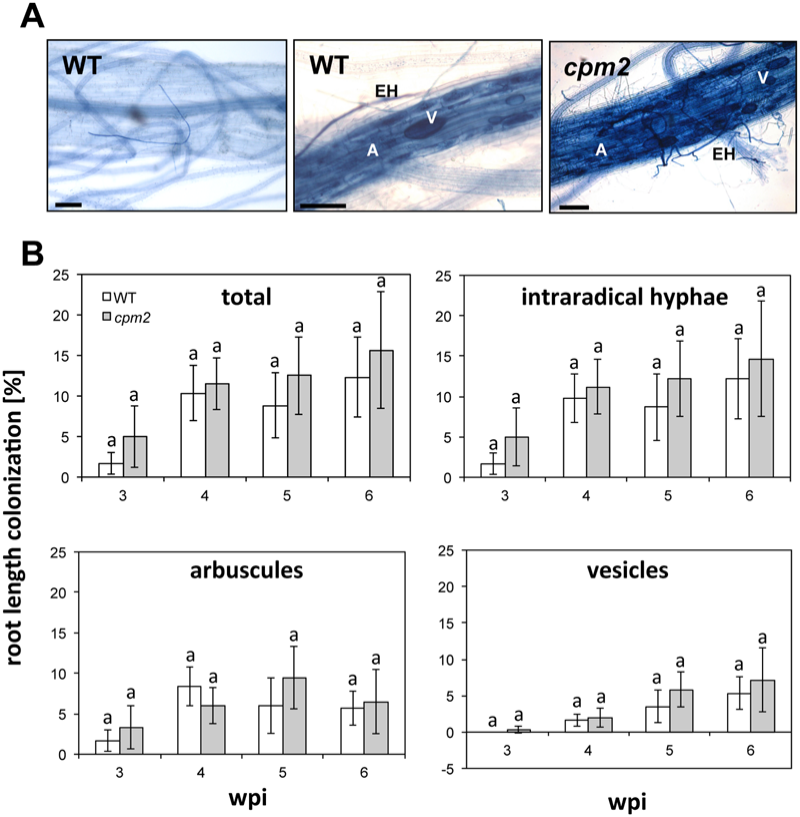
Colonization kinetics of wild-type vs. *cpm2* mutant roots. **A**) Colonization of wild type and *cpm2* roots with *Rhizophagus irregularis* at six weeks post inoculation (wpi). Fungal structures were stained by trypan blue. A non-colonized wild type root is shown on the left for comparison. A, arbuscule; EH, extraradical hypha; V, vesicle; size bars, 100 μm. **B**) Plants were inoculated with *Rhizophagus irregularis* and percent root length colonization was scored with a modified gridline intersect method after trypan blue staining. Means and standard errors from three biological replicates each consisting of a pool of two root systems are shown. Each replicate was represented by 20 root pieces of 2 cm length. As shown by letters no significant differences were found between *cpm2* and wild-type (ANOVA, posthoc Tukey; p≤0.05, n = 24). F_7, 16_(total) = 1.257; F_7, 16_(int. hyphae) = 1.153, F_7, 16_(arbuscules) = 1.067, F_7, 16_(vesicles) = 1.938.

Colonization of *cpm2* and wild type with *Rhizophagus irregularis* was quantified at 3, 4, 5 and 6 weeks post inoculation (wpi). At none of the time points colonization levels of *cpm2* roots differed from the wild-type and fungal structures had a wild-type like morphology (Fig 1) indicating that the lack of JA did not influence AM colonization of *cpm2*. This was reminiscent of *Nicotiana attenuata* lines silenced for the JA-biosynthesis gene LIPOXIGENASE 3 (LOX3) and the JA-perception component CORONATINE INSENSITIVE 1 (COI1) in which AM colonization levels were similarly not affected [28].

**Fig 2:**
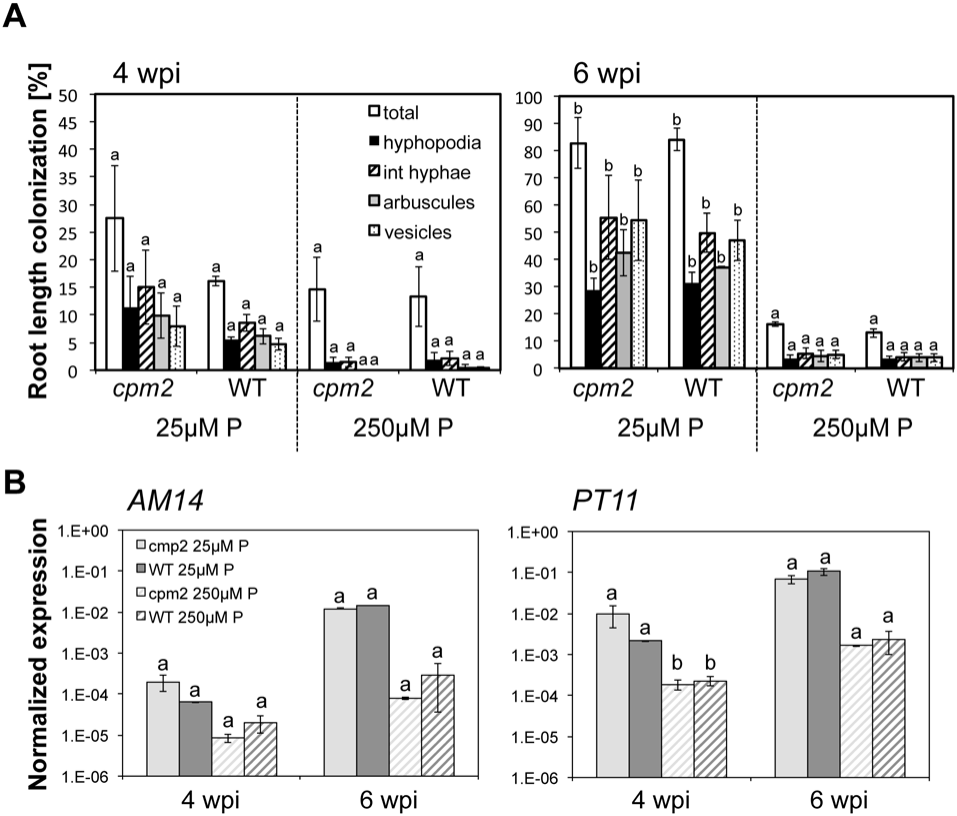
Phosphate-dependent colonization of *cmp2* and WT. **(A)** Percent root length colonization of *cpm2* and wild type roots with *Rhizophagus irregularis* at four (left) and six (right) weeks post inoculation (wpi) after fertilization with 25μM or 250μM phosphate. Root length colonization was determined by a modified gridline intersect method. Note the difference in scale between four and six wpi. Int hyphae, intraradical hyphae. Means and standard errors of six biological replicates are shown. Each replicate was represented by 20 root pieces of 2 cm length. Significant differences (ANOVA, posthoc Tukey; p≤0.05, n = 48) are indicated by different letter for comparisons within the same category (*i*. *e*. fungal structure). F(total)_7,36_ = 44.188, F(hyphopodia)_7,36_ = 21.993, F(int. hyphae)_7,36_ = 24.046, F(arbuscules)_7,40_ = 30.634, F(vesicles)_7,40_ = 28.588. **B**) Expression of arbuscule marker genes *AM14* and *PT11* at four and six wpi in *cmp2* (light grey) and wild-type (dark grey) roots colonized by *Rhizophagus irregularis* and fertilized with either 25 μM (filled bars) or 250 μM (hashed bars) phosphate. Different letters indicate values that were significantly different (p≤0.05, n = 24) as determined by an ANOVA with posthoc test Tukey (F(*AM14*)_7,16_ = 1.676; F(*PT11*)_7,16_ = 21.499).

It has been speculated previously that the requirement of JA for full establishment of AM symbiosis was dependent on the degree of mutualism between plant and fungus [22]. As phosphate delivery by the fungus is an important component of mutualism we addressed whether the requirement of JA-biosynthesis for colonization would differ when the efficiency of mycorrhizal phosphate-uptake pathway was modulated. To address this we fertilized *Rh*. *irregularis* inoculated *cpm2* and wild-type plants with two different phosphate concentrations (25μM and 250 μM). AM colonization was quantified at four and six wpi. Colonization of roots that were fertilized with 25 μM phosphate increased significantly (ANOVA, posthoc Tukey, p≤0.05) between four and six wpi, while in roots fertilized with 250 μM no significant increase in colonization level was observed over time (Fig 2A), indicating that 250 μM phosphate was sufficient to partially suppress further colonization in rice. Sufficient phosphate nutrition has been repeatedly observed to suppress AM colonization in several different plant species [29]. Importantly, percent root length colonization was not significantly different between *cpm2* and wild-type roots at both time points and phosphate concentrations. Therefore, JA was not required for colonization at the tested phosphate concentrations. The expression level of two marker genes *AM14* and *PT11* that indicate active arbuscules [30] confirmed that there was no difference in colonization with active arbuscules between *cpm2* and wild-type (Fig 2B). However, although the mean expression level of both genes appeared to be lower at higher phosphate concentrations, a phosphate-dependent significant expression difference (ANOVA, posthoc Tukey; p≤0.05) for both phenotypes could only be detected for *PT4* expression at four wpi.

**Fig 3:**
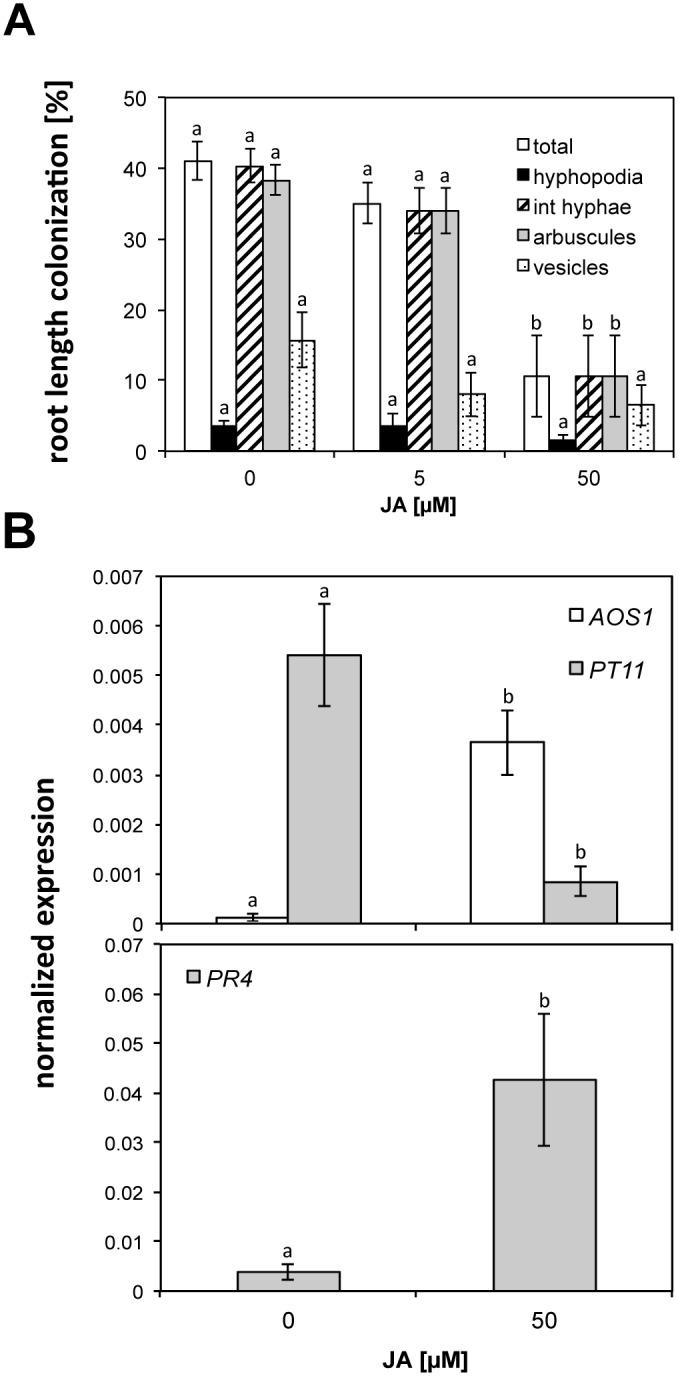
Influence of JA application on AM colonization. Plants were inoculated with *Rhizophagus irregularis* and watered twice weekly with 10 ml of jasmonic acid (JA) solution of the indicated concentrations. AM colonization and gene expression were recorded at six wpi. **A**) Influence of 5 and 50 μM JA application to the roots on colonization of wild-type plants. Means and standard errors for three replicate plants are shown. Different letters indicate significantly different values (p≤0.05, n = 9) for comparisons within the same category (*i*. *e*. fungal structure) as determined by an ANOVA with posthoc test Tukey. F(total)_2,6_ = 15.87; F(hyphopodia)_2,6_ = 1.0; F(int. hyphae)_2,6_ = 14.878; F(arbuscules)_2,6_ = 13.682; F(vesicles)_2,6_ = 2.857. Int. hyphae, intraradical hyphae. **B**) Real time RT-PCR based expression in mycorrhizal roots of the AM marker gene *PT11*, the JA response gene *Allene Oxide Synthase 1* (*AOS1*) and the defense marker gene *Pathogenesis Related Protein 4* (*PR4*) in response to application of 50 μM JA. Gene expression values were normalized to the expression of the constitutive gene *CYCLOPHILIN2* and represent means of three biological replicates with standard errors. Different letters indicate significant differences for the same gene between treatments as determined by an ANOVA, posthoc Tukey (F(*AOS1*)_1,4_ = 28.764; F(*PT11*)_1,4_ = 18.088; F(*PR4*)_1,4_ = 8.282).

Since JA-biosynthesis and—sensitivity are dependent on the light environment [31–34] we considered that under the light conditions applied in our phytochamber (12/12h day night cycle, 400μmoles m^-2^ s^-1^) JA-levels or—sensitivity in the wild type might be too low to reveal a significant difference in AM colonization by *Rh*. *irregularis* between wild-type and the JA-deficient mutant *cpm2*. Thus, we sought to modulate AM colonization by treating wild-type rice plants with exogenous JA. Rice plants inoculated with *Rh*. *irregularis*, were treated with 5 μM and 50 μM jasmonic acid-supplemented fertilizer solution twice weekly. Treatment of 5 μM JA did not alter the root colonization level at six wpi as compared to the solvent-treated control. However, treatment with 50 μM JA reduced colonization by almost three quarters. This reduction was significant (ANOVA, posthoc test Tukey; p≤0.05) for the parameters total colonization, intraradical hyphae and arbuscules (Fig 3A). Consistently, real time RT-PCR based transcript accumulation of the AM marker gene *PT11*, the expression of which is well correlated with the number of arbuscules [30], was also suppressed by treatment with 50 μM JA (Fig 3B). To examine whether the roots had responded to the JA treatment we used the JA-marker gene *Allene Oxide Synthase 1* (AOS1 [35]). This gene encodes an important enzyme of the JA biosynthesis pathway and is subject to feed forward regulation by JA. Accumulation of the JA-marker transcripts had increased (Fig 3B) confirming that the roots had responded to the JA treatment.

AM development requires a signaling cascade called common SYM pathway in which nuclear calcium spiking acts as a second messenger [12,36]. JA has been shown to suppress calcium spiking [37]. Thus, reduced AM colonization after treatment with high JA concentrations might be caused by perturbed common SYM signal transduction. Another possibility or a result of suppressed common SYM signaling might be induction of defense responses, which might have counteracted colonization, as suggested by Herrera-Medina *et al*. [21]. To examine induction of defense signaling the defense marker gene *pathogenesis related protein 4* (*PR4*) was used, which had previously been shown to be induced during root invasion by the hemibiotrophic pathogenic fungus *Magnaporte oryzae* and the nectrotrophic pathogenic fungus *Fusarium moniliforme* [38]. *PR4* transcripts accumulated in mycorrhizal roots treated with 50 μM JA to a level that was more than one order of magnitude higher than in non-treated mycorrhizal roots. This was suggestive of defense induction by treatment with 50 μM JA.

## Conclusions

In this study we show that in contrast to tomato and *Medicago truncatula* but similar to *Nicotiana attenuata* a deficiency in JA-biosynthesis does not affect AM colonization of rice roots. Addition of high concentrations of JA to roots decreased AM colonization. This might be caused by JA-mediated induction of defense mechanisms—as suggested by transcriptional activation of *PR4—*that counteracted compatibility. It will now be interesting to determine how symbiosis with other AM fungal species would depend on JA [39] and how AM colonization of rice would be influenced by pathogenic JA-inducing interactions for example with herbivores, nematodes or pathogenic fungi and which role JA-signaling in such tri- or multi-trophic interactions plays. In another monocotyledon barley JA-biosynthesis was induced by AM colonization. It remains to be determined whether—similar to rice—also in other monocotyledons JA-biosynthesis is dispensable for AM colonization.

## Materials and Methods

### 1. Biological material, growth conditions and quantification of root length colonization

*Oryza sativa* ssp. *japonica* cv. Nihonmasari wild-type and *cpm2* mutant plants [26] were inoculated with *R*. *irregularis*, grown, fertilized and harvested at six weeks post inoculation as described previously [30]. For fertilization of plants with different phosphate concentrations half Hoagland solution was used. This solution was supplemented with either 25 μM or 250 μM phosphate and the potassium concentration was adjusted using KCl. Sequestren rapid (Syngenta) was used as an iron source instead of Fe-citrate [23]. Plants were fertilized twice a week with 10 ml per plant of this solution and watered once a week with 10 ml per plant of de-ionized water. Root length colonization was quantified with a modified gridline-intersect method as described [30].

### 2. Treatment with jasmonic acid

For JA-treatment the fertilizer solution [30] was supplemented with 5 μM or 50 μM jasmonic acid (Sigma-Aldrich, St Louis, USA) solubilized from a stock solution in 100% methanol. All plants were treated with the same amount of methanol and were watered with 10 ml of JA or control solution twice weekly.

### 3. RNA extraction, cDNA synthesis and qPCR

RNA extraction, cDNA synthesis and qPCR were performed as described previously [30]. Primers for the constitutive gene *CYCLOPHILIN* and the AM marker genes *AM14*, *PT11* and *Pathogenesis Related Protein 4* (*PR4*) were taken from [38]. For qPCR amplification of *Allene Oxide Synthase 1* (*AOS1*) transcripts the primers: F_ GGAAGGGGAGATGCTGTTC; R_ GGAGTCGTATCGGAGGAAGA were used.

### 4. Statistical analysis

Probability values were calculated by an ANOVA with a posthoc Tukey test using the program SYSTAT 10 according to the manufacturers instructions.
